# Editorial: Positive higher education: empowering students through learning and wellbeing

**DOI:** 10.3389/fpsyg.2026.1850140

**Published:** 2026-05-15

**Authors:** Maggie Yue Zhao, Ronnel B. King, Ulrike Lichtinger

**Affiliations:** 1 Teaching and Learning Innovation Centre, The University of Hong Kong, Pokfulam, Hong Kong SAR, China; 2 Department of Curriculum and Instruction, The Chinese University of Hong Kong, Hong Kong, Hong Kong SAR, China; 3 IU Internationale Hochschule GmbH, Erfurt, Germany

**Keywords:** higher education, learning, positive psychology, positive education, wellbeing

As the landscape of higher education evolves, there is a growing recognition that a university's role extends beyond cognitive development and academic success. Recently, students' wellbeing and holistic flourishing have moved from peripheral concerns to central organizing principles of higher education (e.g., Curren et al., 2024; Kristjánsson and VanderWeele, 2025; OECD, 2025). This paradigm shift has been profoundly heightened by the COVID-19 pandemic, which precipitated a global surge in psychological distress and uncertainty regarding academic success among university students (Plakhotnik et al., 2021).

In this context, wellbeing, or more broadly, flourishing, implies that students are not merely surviving the rigors of higher education but are thriving in psychological, emotional, and social domains as well. Learning and wellbeing are inherently synergistic and mutually reinforcing processes, each shaping and amplifying the other. As universities prepare students for an increasingly unpredictable global landscape, fostering wellbeing is no longer a supplementary goal but an institutional imperative. Cultivating these qualities aligns with a broader, more expansive vision of higher education, one that seeks to nurture individuals who are not only academically capable but also empowered to meaningfully engage with an ever-evolving world.

To address this dual imperative of learning and wellbeing, an emerging paradigm, positive higher education, has garnered substantial scholarly attention. Rooted in the science of positive psychology and positive education (Seligman and Csikszentmihalyi, 2000; Seligman et al., 2009), this field examines prototypical constructs such as resilience, social-emotional skills, character strengths, and positive emotions among others (e.g., Allen et al., 2024; King et al., 2024; Wang et al., 2025a,b; Xie et al., 2023).

Positive higher education covers a broad array of ideas and frameworks. In this sense, positive higher education is best understood not as a prescriptive program or an isolated model, but rather as an overarching perspective, a strengths-based lens through which educational systems can be reimagined. While the initial growth of positive education occurred predominantly within primary and secondary schools, there are now promising signs of its integration into higher education. This Research Topic, *Positive Higher Education: Empowering Students through Learning and Wellbeing*, aims to explore this burgeoning, multidisciplinary field in a comprehensive and timely manner. By bringing together scholarship at the intersection of higher education and positive education, this collection seeks to encourage universities and other tertiary institutions to harness more effectively the synergy between learning and wellbeing.

The collection of articles in this Research Topic provides both empirical evidence and theoretical insights that advance positive higher education. Drawing on diverse methodologies, ranging from quantitative approaches and scoping reviews, these contributions collectively advance our understanding of how psychological resources, social connectivity, and pedagogical innovations can be meaningfully integrated into higher education environments.

## Psychological resources

A major strand of scholarship centers on the cultivation of internal psychological resources that sustain wellbeing, motivation, and academic success. Across the studies, psychological capital, character strengths, self-efficacy, resilience, and self-regulation emerge as core personal assets through which students interpret challenge, maintain commitment, and engage productively with university life (Alismail et al.; Bagdžiūnienė et al.; Cai and Meng; García-Álvarez et al.; Li, C.; Li, H.; Ye et al.). These studies collectively suggest that wellbeing in higher education is not only shaped by external support structures, but also by the development of durable internal capacities that enable students to navigate demanding academic environments.

Within this body of work, psychological capital, encompassing hope, optimism, resilience, and self-efficacy, emerges as a particularly influential construct, linking students' academic satisfaction to their motivation, self-directed learning, and broader sense of agency. Character strengths such as hope and persistence further deepen this picture, indicating that students' beliefs about their own capabilities are closely connected to both general and academic functioning.

Academic resilience, likewise, is framed not simply as a fixed personal trait, but as a dynamic capacity shaped by the interplay between individual efficacy and study-related conditions. Viewed alongside this emphasis on internal strengths, the scoping review of wellbeing interventions in U.S. colleges highlights the potential of approaches such as mindfulness and cognitive-behavioral strategies to reduce student stress, while also drawing attention to conceptual ambiguity and the limited long-term institutionalization of such efforts (Zhao et al.). Taken together, these contributions underscore that the cultivation of psychological resources remains central to positive higher education, even as important questions persist regarding conceptual coherence, scalability, and sustainable implementation.

## Social connectivity

A second cluster of contributions demonstrates that wellbeing in higher education is profoundly relational. The university experience is shaped not only by what students bring with them internally, but also by the quality of the social environments in which they learn, belong, and develop. Across this issue, social support, belonging, peer and teacher relationships, and group attachment emerge as critical dimensions of student wellbeing and engagement (Dost; Hanimoglu; Li et al.; Wang, Lu et al.). The evidence in this area points to several consistent patterns. Social support is associated with greater life satisfaction and resilience, whereas loneliness, reduced belonging, and academic anxiety are linked to weaker coping self-efficacy and lower participation. Such findings emphasize that the social dimensions of university life are not peripheral to wellbeing; they are among its most consequential conditions.

This work also shows that support operates at multiple levels within the university environment. Teacher and peer support are associated with academic engagement, both directly and through basic psychological need fulfillment and autonomous motivation (Li et al.). Overall, these contributions suggest that student wellbeing and engagement are shaped not only by individual attributes and resources, but also by the quality of the relational contexts in which learning takes place.

## Pedagogy and learning environment

A third and closely related area of contribution concerns the immediate educational environment itself: pedagogy, assessment, teaching quality, and the wellbeing of those who educate and supervise students. This body of work highlights that learning and wellbeing are deeply intertwined, and that the everyday architecture of university life, clear expectations, constructive feedback, meaningful relationships, and emotionally supportive teaching practices, plays a decisive role in shaping student outcomes (Asare; Cheng et al.; Wammerl and Lichtinger; Wang, Jaafar et al.; Xu et al.; Zhao and Hua).

Across the collection, students' university learning experiences are shown to predict not only academic engagement, but also hedonic, eudaimonic, and social wellbeing. Internal dimensions of learning, such as cognitive, social, and value development, appear especially powerful, while external dimensions, including teaching quality, goals and standards, and constructive feedback, also contribute substantially to student flourishing. These findings underscore that pedagogy is not merely a mechanism for knowledge transmission, but a formative developmental context in which students construct meaning, identity, and agency, interpret challenge, derive meaning and build confidence.

Emotion also emerges as a central pedagogical focus. Positive emotional experiences broaden students' cognitive and motivational resources, enhancing engagement, life satisfaction, and persistence. This has important implications for instructional design, particularly in relation to innovative approaches such as gamification, growth mindset interventions, and peer-assisted learning. Such pedagogical strategies do more than improve performance in narrow academic terms; they create learning environments that can increase enjoyment, focus, teamwork, self-regulated learning, and confidence in the face of academic difficulty. The evidence presented here suggests that positive pedagogy is most effective when it addresses both the intellectual and emotional dimensions of student experience.

Further, the issue extends the conversation beyond classroom teaching to include supervision, leadership, and educator wellbeing. The emotional climate created by supervisors and teacher educators has significant implications for student wellbeing, particularly in advanced and professionally oriented forms of study (Mavrogalou-Foti et al.; Gilar-Corbi et al.). Ambiguity in supervision, burnout risk, and weak professional support emerge as threats to wellbeing, while resilience, self-efficacy, and supportive leadership function as protective factors. These contributions make clear that the wellbeing of students is inseparable from the wellbeing, relational competence, and leadership of those who teach, mentor, and prepare the next generation of educators.

## Advancing positive higher education

In conclusion, this Research Topic provides compelling empirical evidence that integrating positive psychology into higher education can generate wide-ranging benefits. The findings extend existing scholarship and offer fresh insight into the mutually reinforcing relationship between learning and wellbeing. From a pedagogical perspective, the studies highlight academic practices, teaching approaches, and learning environments that can promote student flourishing. From a psychological perspective, they deepen understanding of how internal resources, such as psychological capital, interact with external resources, including peer and supervisory support, to shape student experiences and outcomes.

In practice, these findings can inform the development of targeted policies and holistic interventions within higher education institutions. Prioritizing the wellbeing of both students and staff requires strengthening support services and embedding positive education perspectives/approaches into educational and professional development initiatives. More broadly, flourishing can be fostered through curriculum design that incorporates character development, clear academic expectations, and inclusive pedagogical practices.

Overall, the studies in this Research Topic underscore the importance of strategies that do not just put the onus on the individual student but recognizes the relational and institutional contexts within which wellbeing occurs. As higher education continues to expand and evolve globally, it is hoped that this body of work will encourage educators, administrators, and policymakers to treat wellbeing not as an adjunct to academic achievement, but as a core condition for meaningful and sustainable learning. In doing so, universities can better fulfill their broader developmental mission: to cultivate resilient, engaged, and flourishing individuals who are equipped to contribute meaningfully to society.
